# Thought Control Ability Is Different from Rumination in Explaining the Association between Neuroticism and Depression: A Three-Study Replication

**DOI:** 10.3389/fpsyg.2017.00838

**Published:** 2017-05-31

**Authors:** Feng-Ying Lu, Wen-Jing Yang, Qing-Lin Zhang, Jiang Qiu

**Affiliations:** ^1^Faculty of Psychology, Southwest UniversityChongqing, China; ^2^Key Laboratory of Cognition and Personality (SWU), Ministry of Education, Southwest UniversityChongqing, China

**Keywords:** neuroticism, depression, thought control, rumination, mediation

## Abstract

Neuroticism is the most common vulnerability factor of depression. However, the mechanism underlying this vulnerability is still unclear. Previous studies suggested that rumination intensifies the negative effect of neuroticism on depression. However, whether cognitive control could explain the association between neuroticism and depression remains unclear to date. Therefore, this study evaluated the indirect effects of rumination and thought control on the relationship between neuroticism and depression. Seven self-report measures were employed among healthy and main depression disorder (MDD) participants. Three studies were used to examine the hypotheses. Results of the three studies showed significant correlations among neuroticism, rumination, thought control, and depression. Rumination mediated the link between neuroticism and depression among healthy young adults, and this finding replicated previous studies. This study provided new evidence that thought control mediates the association between neuroticism and depression in both healthy and MDD populations. In conclusion, rumination increases neuroticism risk for depression, but high-level thought control decreases the effect of neuroticism on depression. This study may serve as a reference to develop effective and focused interventions for MDD patients.

## Introduction

Main depression disorder (MDD) is mainly characterized by depressive mood and anhedonia (Disner et al., 2011). Approximately 80% of depressed individuals recurrently experience episodes of depression (Boland and Keller, 2009). The prevalence of MDD is high, such that it is considered to be the most burdensome disease in the 21st century (World Health Organization, 2008). In consideration of these alarming figures and trends, further identification of the risk factors and mechanisms that contribute to the elevation of depression is necessary to develop effective approaches for preventing episodes of depression.

Neuroticism is a core personality trait highly relevant to psychopathology, especially depression disorder (Roelofs et al., 2008; Kotov et al., 2010; Ormel et al., 2013). High levels of neuroticism indicate depressive symptoms in both youth (Wängqvist et al., 2015) and adults (Newtonhowes et al., 2015). Longitudinal and cross-sectional studies revealed a significant association between neuroticism and depressive symptoms among clinical and nonclinical participants (Boyce et al., 1991; Dunkley et al., 2009; Loey et al., 2014; Paulus et al., 2015). Previous research interpreted that highly neurotic individuals are sensitive to emotional stimuli that encompass the tendencies to experience negative effects, including depression, when they encounter life stressors (Kendler et al., 2004). Despite the fact that neuroticism is a risk factor for depression, the cognitive mechanism underlying neuroticism and depression remains unclear (Barnhofer and Chittka, 2010).

Rumination is defined as repetitively thinking about one’s negative emotional experience in terms of its causes, consequences, and situational factors (Nolen-Hoeksema, 1998). Rumination is a major cognitive risk factor for depression (Koster et al., 2011). In specific, rumination is associated with depressive symptoms (Robinson and Alloy, 2003); the onset (Nolenhoeksema, 2000), severity (Just and Alloy, 1997), and duration (Mclaughlin and Nolenhoeksema, 2011) of depression; and recovery from depression (Schmaling and Dimidjian, 2002). Furthermore, rumination partially mediates the association between neuroticism and depression (Muris et al., 2005; Roelofs et al., 2008). Previous results suggested that highly neurotic individuals are sensitive to negative information when meted with stressors (Kendler et al., 2004). Rumination compels individuals to recall past negative events repeatedly (Bar et al., 2007). This cognitive processing intensifies the negative effect of neuroticism on depressive symptoms.

In contrast to rumination, thought suppression is a self-control thinking style employed by individuals to keep unwanted and intrusive thoughts out of mind (Wells and Davies, 1994). Thought suppression is important for a goal-directed control of thoughts and behaviors (Botvinick et al., 2001; Aron et al., 2004). It eliminates disruptive thoughts and gives focus on productive activities. High-level thought control is related to high working memory capacity and fluid intelligence (Brewin and Beaton, 2002). Previous studies suggested that suppressing intrusive memories or thoughts could lower the level of neuroticism (Luciano et al., 2005) and the symptoms of depression (Joormann et al., 2007). However, whether high-level thought suppression could reduce the negative effect of neuroticism on depressive symptoms has yet to be determined.

Rumination and cognitive control are critical cognitive processes in depressed individuals. Individuals with MDD have negative views about oneself, the world, and the future (Beck and Alford, 2009). These individuals are particularly sensitized to negative aspects of information from the environment (Rock et al., 2014). Large negative information is encoded into the working memory, which exhibits depressive rumination (Spasojević and Alloy, 2001) and then leads to depression (Disner et al., 2011). To some degree, cognitive control deficits contribute to this working memory processing (Gotlib and Joormann, 2010; Joormann and Gotlib, 2010). Hence, depressed individuals demonstrate impaired cognitive control (Reppermund et al., 2009; Rock et al., 2014). For example, depressed individuals show no significant difference from non-depressed individuals in recalling structured information but encounter problems in recalling unconstrained information; this result can be ascribed to the fact that recalling unconstrained information requires goal-oriented behavior and cognitive flexibility requires cognitive control (Hertel, 2004). Depressed individuals experience difficulties in inhibiting irrelevant information from cognitive processing and in removing irrelevant information from the working memory (Gotlib and Joormann, 2010). In addition, a significant relationship exists between rumination and directed forgetting using emotional stimuli (Dieler et al., 2014). Individuals with a low level of rumination are significantly more prone to suppression-induced forgetting than those with a high level of rumination (Fawcett et al., 2014). These findings demonstrate that rumination and cognitive control are relatively contrary cognitive processes in depression. Thus, exploring the effects of rumination and thought control on the relationship between neuroticism and depressive symptoms is significant to develop effective interventions for MDD patients.

According to a previous review, neuroticism is a personal risk trait for depression (Newtonhowes et al., 2015; Wängqvist et al., 2015). However, the cognitive mechanism underlying the association between neuroticism and depression is still unclear. Rumination enhances depressive symptoms in highly neurotic individuals (Roelofs et al., 2009). Meanwhile, high-level cognitive control decreases depression (Gotlib and Joormann, 2010) and is negatively related to neuroticism (Luciano et al., 2005). However, whether thought control could explain the association between neuroticism and depressive symptoms remains unclear. Thus, the present study investigated the cognitive mechanism underlying the association between neuroticism and depression while simultaneously considering rumination and thought control as mediating variables. This study hypothesized that (1) significant correlations exist among neuroticism, rumination, thought control, and current depressive symptoms, and that (2) both rumination and thought control mediate the relationship between neuroticism and current depressive symptoms. Three studies were used in the present study to investigate these hypotheses. Study 1 explored the association between neuroticism and depressive symptoms while simultaneously considering rumination and thought control as mediating variables among healthy young adults. To attain reliable results, Study 2 sought to replicate the findings from study 1. Study 3 examined whether the results of nonclinical young adults could be further extended in a clinical group of adults with MDD.

## Study 1

The main purpose of Study 1 is to examine the association between neuroticism and depressive symptoms while simultaneously considering rumination and thought control as mediating variables among healthy young adults.

### Methods

#### Participants and Procedures

A total of 74 healthy university students (37 females and 37 males; mean age = 22.17, *SD* = 1.12, age range = 20–27 years old) participated in this study. The Structured Clinical Interview for DSM-IV Axis I disorders was used to diagnose psychiatric disorders (Wittchen, 1997). Healthy participants had neither previous or concurrent psychiatric disorders nor a history of drinking, smoking, or drug addiction. The present study is a part of an ongoing project that investigates the association among brain imaging, creativity, and mental health (Li, 2014; Wei et al., 2014).

Participants were recruited by advertisements on bulletin boards or campus network. Most of the participants were from Southwest University, Chongqing, China. The participants came into the laboratory to complete the survey. They were informed that they were voluntarily involved in the study. After providing written informed consent, the participants were asked to respond to a series of psychological tests. Questionnaires were completed anonymously to ensure confidentiality. The Research Ethics Committees of the Brain Imaging Center of Southwest University approved this project.

#### Measures

##### Neuroticism

Neuroticism is one subscale of the neuroticism, extraversion, openness to experience personality inventory-revised (NEO-PI-R) (Costa and Mccrae, 1992). Neuroticism includes a 42-item self-report and reflects six-domain neurotic personality traits, such as worry, anger, discouragement, self-consciousness, impulsivity, and vulnerability. The participants were asked to read a statement that reflects personal characteristics and then to select the appropriate response on a five-point Likert scale ranging from 1 (strongly disagree) to 5 (strongly agree). NEO-PI-R is widely used among the Chinese (Luo et al., 2016). This study focused on neuroticism; thus, the neuroticism subscale was used for further analysis. Cronbach’s alpha for neuroticism was 0.90 in Study 1.

##### Thought control

The Thought Control Ability Questionnaire (TCAQ) was used to assess the cognitive control ability of the participants (Luciano et al., 2005). TCAQ is a 25-item self-report questionnaire used to measure the ability of an individual to control unwanted and intrusive thoughts (Williams et al., 2010). TCAQ has a high test–retest reliability and good internal consistency (Luciano et al., 2005). The participants were required to rate on a five-point Likert scale, from 1 (completely disagree) to 5 (completely agree), the extent to which they agree with each statement. TCAQ is a unifactorial construct inventory with scores ranging from 25 to 125. High scores reflect greater thought control ability over intrusive memories. TCAQ scores could predict individual differences in the successful suppression of unwanted thoughts (Luciano et al., 2005). Cronbach’s alpha for TCAQ was 0.89 in Study 1.

##### Rumination

The Short Ruminative Responses Scale (SRRS) is a 10-item self-report questionnaire, which is a revised short version of the Ruminative Response Scale (Han, 2009; Zhang and Xu, 2010). SRRS includes two aspects of rumination, namely, sensitive rumination and assessment rumination. SRRS has excellent reliability and validity (Treynor et al., 2003) in Chinese college students (Zhang and Xu, 2010). SRRS describes how individuals respond to depressive moods, including symptom attention (e.g., I am thinking of how difficult it is to focus on something.), self-focused attention (e.g., Why am I so reactive?), and causes and results (e.g., I don’t think I can do a job if I can’t get rid of this situation.). The participants responded on a four-point Likert scale (1 = almost never, 2 = sometimes, 3 = often, 4 = almost always). High scores on SRRS reflect serious rumination. Cronbach’s alpha for SRRS was 0.90 in Study 1.

##### Depressive symptoms

Beck Depression Inventory-Second Edition (BDI-II) is a 21-item self-report questionnaire that is widely used to measure depressive symptoms (Beck et al., 1996). BDI-II was developed on the basis of DSM-IV. The participants were required to rate their feelings over the past two weeks on a 0–3 scale. High scores indicate serious depressive symptoms. The cutoff scores of BDI-II reflect different levels of depression: 0–13, minimal; 14–19, mild; 20–28, moderate; and 29–63, severe (Beck et al., 1996). The mean score of BDI-II was 6.27 (*SD* = 8.89). The range was 0–43 in the current study. Cronbach’s alpha for BDI-II was 0.92 in Study 1.

#### Statistical Analysis

Statistical analyses were performed using the statistical software SPSS16.0. Pearson’s correlation coefficient and two dependent-sample *t*-tests were used to analyze the main variables in this study. Indirect effect (IE) analysis was conducted using the indirect macro design for SPSS (Preacher and Hayes, 2008). This macro used bootstrapped sampling to estimate the indirect mediating effect. To adequately calculate for multiple comparisons and a small sample size, 5000 bootstrapped samples were drawn and 95% bootstrap confidence intervals (CI) were reported. On the basis of the analysis of Preacher and Hayes (2008), the upper and lower CIs did not include zero, indicating a significant IE of the independent variable (X) on the dependent variable (Y) through the mediators (M). Path *a* represents the effect of X on M. Path *b* represents the effect of M on Y. Path *c* represents the direct effect of X on Y. Path *c’* represents the effect of X on Y controlling for M. The IE of X on Y through M is the product of *a* and *b* paths, that is, IE = *ab, c* = *c’* + *ab.* In the present study, neuroticism is the independent variable, depressive symptoms are the dependent variables, and thought control ability and rumination are the mediators. The multiple mediation model was used to estimate the parameters of all variables simultaneously in the same model. This model not only decides whether or not an IE exists but also determines the relative magnitudes of the specific IEs associated with all mediators (Preacher and Hayes, 2008).

### Results

#### Descriptive Statistics

The results of descriptive statistics are shown in **Table 1**. The main variables indicated no significant differences between males and females in this study.

**Table 1:** Descriptive statistics in Study 1 (*M, SD*).

Variables	Total (74)	Males (37)	Females (37)	*t*
Age	22.19 (1.12)	19.36 (1.13)	19.53 (1.13)	–0.65
Neuroticism	2.83 (0.42)	2.83 (0.35)	2.83 (0.49)	0.09
Rumination	2.08 (0.49)	2.18 (0.47)	1.93 (0.49)	1.77
TCAQ	3.22 (0.48)	3.17 (0.47)	3.27 (0.49)	–0.81
BDI	0.30 (0.42)	0.49 (0.08)	0.28 (0.34)	0.44

*M, mean; SD, standard deviation; Neuroticism, the mean scores of one subscale of the NEO Personality Inventory-Revised; Rumination: the mean scores of Short Ruminative Responses Scale (SRSS); TCAQ: the mean scores of Thought Control Ability Questionnaire; BDI: the mean scores of Beck Depression Inventory-Second Edition (BDI-II). Similar descriptive terms are used hereinafter.*

#### Correlations

Age and sex were added as covariates into the correlation model. Results showed significant associations among the main variables (**Table 2**). In specific, neuroticism was positively associated with rumination and depression but negatively associated with thought control. Rumination was positively associated with depression but negatively associated with thought control. Thought control was negatively associated with depression. That is, strong correlations existed among neuroticism, rumination, thought control, and depressive symptoms among healthy young adults.

**Table 2:** Correlation matrix in Study 1.

Variables	Neuroticism	Rumination	TCAQ
Neuroticism	–		
Rumination	0.64^∗∗∗^	–	
TCAQ	–0.58^∗∗∗^	–0.35^∗∗∗^	–
BDI	0.43^∗∗∗^	0.49^∗∗∗^	–0.24^∗^

*^∗^*P* ≤ 0.05, ^∗∗^*P* ≤ 0.01, ^∗∗∗^*P* ≤ 0.001, two tailed test.*

#### Mediation Results

**FIGURE 1:**
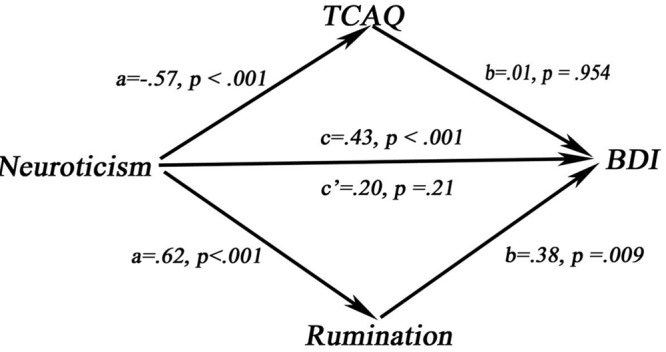
The mediating model.

The multiple mediation model was used to reveal the IE of rumination and thought control on the relationship between neuroticism and depressive symptoms while considering sex and age as covariates. Results showed that rumination mediated the relationship between neuroticism and BDI (IE = 0.24, SE = 0.09, CI = 0.097–0.469). Although correlations among neuroticism, TCAQ, and BDI were detected, the TCAQ mediating effect was not significant between neuroticism and BDI (IE = –0.004, SE = 0.09, CI = –0.166–0.181). In this multiple model, the adjusted *R*^2^ = 0.21, *p* = 0.006 (**Figure 1**).

### Discussion

In Study 1, although significant associations existed among neuroticism, thought control, and depression, the IE of thought control on the relationship between neuroticism and depression was not significant. Compared with lowly neurotic individuals, highly neurotic are more likely to be depressive when they experience stressful life events (Ormel et al., 2001). Therefore, the associations of main variables while considering life event stressors as covariates in a larger participant size warrant further exploration.

## Study 2

To obtain reliable results from correlation and mediation tests, Study 2 further explored the IEs of rumination and thought control on the relationship between neuroticism and depressive symptoms while considering life event stressors as covariates in a larger participant size.

### Methods

#### Participants and Procedures

A total of 148 healthy university students (112 females and 36 males; mean age = 19.48 years, SD = 1.12, age range = 17–24 years old) from Southwest University, Chongqing, China, participated in this study. These participants are part of our ongoing project to investigate the interactions of gene, brain, and behaviors among university students. All the participants were free from any history of neurological or psychiatric problems. In addition, the procedures and analysis methods were the same as those in Study 1.

#### Measures

##### Stress

The Adolescent Self-Rating Life Events Checklist (ASLEC) is used to assess life event stressors (Liu et al., 1997). ASLEC includes 26 stressful life events that adolescents may have experienced during the past 12 months. The seven types of stressful life events are interpersonal problems, school-related problems, parental problems, punishment, loss, health problems, and adaptation problems. The participants self-rated on a six-point response scale ranging from 0 (did not occur) to 5 (occurred and was extremely stressful). High total scores on ASLEC indicate a large number of stressful life events experienced in the past 12 months. Cronbach’s alpha for ASLEC was 0.97 in the current study.

##### Neuroticism, thought control, and depressive symptoms

NEO-PI-R was also employed in this study to assess the scores of neuroticism, with Cronbach’s alpha of 0.74. TCAQ was used to measure thought control, with Cronbach’s alpha of 0.89. SRRS was utilized to assess rumination, with Cronbach’s alpha of 0.85. BDI was employed to assess the symptoms of depression. The mean score of BDI-II was 7.57 (*SD* = 6.36), the range was 0–31, and Cronbach’s alpha for BDI-II was 0.85.

### Results

#### Descriptive Statistics

The results of descriptive statistics are shown in **Table 3**. The main variables in this study showed no significant differences between males and females. This result was consistent with that in Study 1.

**Table 3:** Descriptive statistics in Study 2 (*M, SD*).

Variables	Total (148)	Males (36)	Females (112)	*t*
Age	19.48 (1.22)	19.36 (1.13)	19.51 (1.25)	–0.58
Neuroticism	3.11 (0.27)	3.19 (0.32)	3.08 (0.25)	1.94
Rumination	1.89 (0.48)	1.86 (0.44)	1.88 (0.50)	–0.14
TCAQ	3.11 (0.49)	3.09 (0.47)	3.11 (0.51)	–0.23
BDI	0.36 (0.30)	0.42 (0.31)	0.34 (0.30)	1.31
Stress	2.34 (1.18)	2.27 (1.01)	2.33 (1.21)	–0.21

*Stress: the mean scores of Adolescent Self-Rating Life Events Checklist.*

#### Correlations

Sex, age, and ASLEC scores as covariates were added into the correlation model. Results showed significant associations among the main variables, consistent with the results in Study 1 (**Table 4**).

**Table 4:** Correlations matrix in Study 2.

Variables	Neuroticism	Rumination	TCAQ
Neuroticism	–		
Rumination	0.23^∗∗^	–	
TCAQ	–0.36^∗∗∗^	–0.24^∗∗^	–
BDI	0.32^∗∗∗^	0.25^∗∗^	–0.43^∗∗∗^

*^∗^*P* ≤ 0.05, ^∗∗^*P* ≤ 0.01, ^∗∗∗^*P* ≤ 0.001, two tailed test, data from Sample 2.*

#### Mediation Results

**FIGURE 2:**
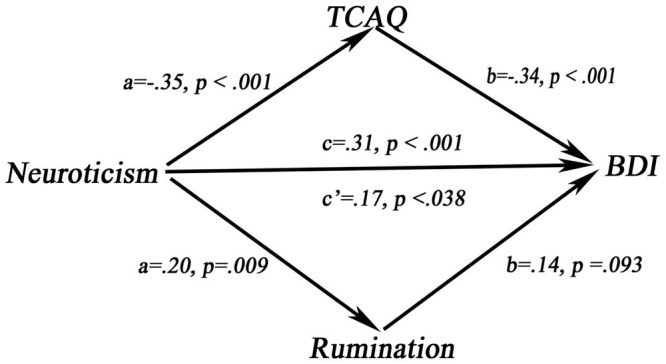
The replication model of mediating effect.

Sex, age, and ASLEC scores were added as covariates into the mediation model. The results of IE analysis showed that rumination mediated the relationship between neuroticism and BDI (IE = 0.03, SE = 0.02, CI = 0.005–0.072). TCAQ played a mediating role between neuroticism and BDI (IE = 0.12, SE = 0.04, CI = 0.068–0.218). In this multiple model, the adjusted *R*^2^ = 0.25, *p* < 0.001 (**Figure 2**). These findings indicate that both rumination and thought control mediated the association between neuroticism and current depressive symptoms among healthy young adults.

### Discussion

Study 1 and Study 2 revealed significant correlations among neuroticism, rumination, thought control, and depressive symptoms occurred. Both rumination and thought control ability partially mediated the association between neuroticism and depressive symptoms among healthy young adults. These results relied on nonclinical individuals. However, whether the current findings could be generalized to clinical participants remains unclear. In addition, the results of the current study were based on a relatively small range of participant age. Whether these results could be found in an extended participant age is unclear. Therefore, further studies should aim at replicating and substantiating the results of the present study in clinical participants.

## Study 3

Study 3 aimed to replicate and further substantiate the results of the previous study in clinical participants with MDD. The results of this study could be used to develop effective and focused interventions for MDD patients.

### Methods

#### Participants and Procedures

Twenty-one MDD adults (16 females, 31.05 ± 9.47 years old, age range: 18–52 years) participated in this study. Patients with MDD were recruited from the Outpatient Department at the First Affiliated Hospital of Chongqing Medical School in Chongqing, China. All subjects were independently diagnosed by two psychiatrists in accordance with the Structured Clinical Interview of DSM-IV. MDD patients met the DSM-IV criteria for main episodes of depression disorder. Detailed information about the participants is provided in a previous study (Liu et al., 2016). The present study was approved by the Research Ethics Committees of the Brain Imaging Center of Southwest University and the First Affiliated Hospital of Chongqing Medical School. The analysis methods were the same as those in Study 1.

#### Measures

##### Neuroticism

Neuroticism is one subscale of the self-report Eysenck Personality Questionnaire (EPQ) (Chen, 1983). This subscale is a 24-item self-report questionnaire that was used to assess the neuroticism of personality for adults. The participants reported their current feeling with “Yes = 1” or “No = 0” according to the description of each sentence. The summary scores of neuroticism were 15.94 ± 6.18 (range: 0–22) in this participant group. Cronbach’s alpha for neuroticism was 0.90 in the present study.

##### Depression

The self-rating depression scale (SDS) was used to assess the level of depressive symptoms among MDD patients. SDS is a 20-item self-reporting scale concerning the affective, cognitive, behavioral, and somatic symptoms of depression (Zung et al., 1965). SDS was designed according to the most common symptoms of depression. The participants rated each item depending on how they felt during the past week. The rating on a four-point Likert scale ranged from 1 (none or a little of the time) to 4 (most or all of the time). The index of SDS is a summary score ranging from 20 to 80. The summary scores were 40.77 ± 11.80 (range: 20–60) in this participant group. Cronbach’s alpha for SDS was 0.91 in the current study.

##### Thought control and rumination

Thought Control Ability Questionnaire was used to assess thought control. TCAQ is suitable for clinical patients (Peterson et al., 2009). The mean scores of TCAQ were 2.80 ± 0.81, and the summary scores ranged from 38 to 117 in this participant group. Cronbach’s alpha for TCAQ was 0.94 in the current study. SRRS was used to assess the rumination variable. SRRS is widely used in clinical participants (Griffith and Raes, 2015). The mean score of SRRS was 2.30 ± 0.71, and the summary scores ranged from 13 to 40 in this participant group. Cronbach’s alpha for SRRS was 0.92 in the current study.

### Results

#### Descriptive Statistics

The results of descriptive statistics are shown in **Table 5**. The main variables in this study showed no significant differences between males and females. The results agreed with those of Study 1 and Study 2.

**Table 5:** Descriptive statistics of main depression disorder (MDD) (*M, SD*).

Variables	Total (21)	Males (5)	Females (16)	*t*
Education years	13.33 (2.80)	12.00 (3.67)	13.75 (2.46)	–1.23
Age	31.05 (9.47)	31.00 (9.67)	31.06 (9.73)	–0.01
Neuroticism	0.66 (0.26)	0.77 (0.08)	0.63 (0.28)	1.09
Rumination	2.30 (0.71)	2.39 (0.51)	2.28 (0.78)	–0.56
TCAQ	2.80 (0.81)	2.62 (0.42)	2.86 (0.90)	–0.31
SDS	2.04 (0.59)	2.09 (0.40)	2.02 (0.65)	0.22

*Neuroticism, the mean scores of one subscale of the Eysenck Personality Questionnaire (EPQ); Rumination, the mean scores of SRSS; TCAQ, the mean scores of Thought Control Ability Questionnaire; SDS, the mean scores of Self-rating depression scale (SDS). Similar descriptive terms are used hereinafter.*

#### Correlations

Sex and age were considered as covariates and were added into the correlation model. Results showed significant associations among the main variables in depressive adults, which were consistent with the results of Study 1 and Study 2 (see **Table 6**).

**Table 6:** Correlations matrix in MDD.

variables	Neuroticism	Rumination	TCAQ
Neuroticism	–		
Rumination	0.76^∗∗^	–	
TCAQ	–0.76^∗∗∗^	–0.87^∗∗^	–
SDS	0.71^∗∗∗^	0.82^∗∗^	–0.95^∗∗∗^

*^∗^*P* ≤ 0.05, ^∗∗^*P* ≤ 0.01, ^∗∗∗^*P* ≤ 0.001, two tailed test.*

Correlation analysis revealed high correlations among the main variables in this study. Thus, collinearity diagnostics was used to check multicollinearity in the multiple linear regression model. TCAQ and rumination scores were added as independent variables, and SDS scores were added as dependent variables. The variance inflation factor (VIF) was used to detect the presence of linear relationships between two independent variables. The VIF associated with TCAQ was 5.63, and the VIF associated with rumination was 4.16. Both values were below 10 (Myers, 1990). Given these figures, multicollenearity bias unlikely existed in the independent variables in this study.

#### Mediation Results

**FIGURE 3:**
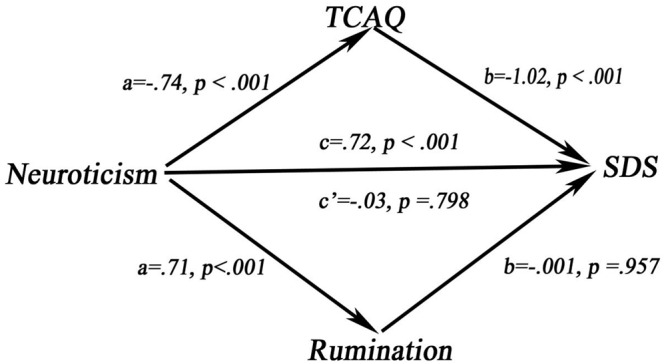
The mediating model in MDD.

Sex and age were added as covariates into the mediation analysis model. Results revealed that TCAQ mediated the relationship between neuroticism and SDS (IE = 0.76, SE = 0.40, CI = 0.26–1.60). However, the IE of rumination on the relationship between neuroticism and SDS was not significant (IE = –0.01, SE = 0.19, CI = –0.36–0.36). In this multiple model, the adjusted *R*^2^ = 0.86, *p* < 0.001 (**Figure 3**). These results demonstrate that thought control but not rumination mediated the association between neuroticism and depression in MDD adults. Thus, thought control is more important than rumination in explaining the relationship between neuroticism and depressive symptoms among MDD patients.

### Discussion

This study was the first to investigate the IE of thought control between neurotic personal trials and depressive symptoms among MDD patients. Results suggested that the association between neuroticism and depression was mediated by thought control. Previous studies revealed that deficits in suppressing negative material processing promote symptoms of depression (Joormann and Quinn, 2014). A meta-analysis revealed cognitive deficits in executive function, memory, and attention relative to controls in patients with depression (Rock et al., 2014). Neuroticism is a critical risk factor for depression (Barnhofer and Chittka, 2010). Highly neurotic individuals display low levels of thought control (Luciano et al., 2005). Thus, the results of this study extend the findings of previous research. The mediation role of thought control between neuroticism and depression demonstrated that improving thought suppression may be a valuable target for future interventions among MDD patients.

## General Discussion

This study is the first to investigate the relationship between neuroticism and current depressive symptoms while considering two relatively contrary cognitive processes, namely, rumination and thought control, as mediating factors in healthy and MDD people. As expected, significant associations were observed among neuroticism, rumination, thought control, and current depressive symptoms. IE analysis showed that rumination mediated the relationship between neuroticism and depression among healthy adults. This finding was consistent with previous results (Muris et al., 2005; Roelofs et al., 2009). The present study provided the first evidence that thought control partially mediates the relationship between neuroticism and current depressive symptoms among healthy and MDD people. Results indicated that rumination and thought control are contrary cognitive processes in explaining the relationship between neuroticism and depressive symptoms.

This study revealed a positive association between rumination and depressive symptoms in both healthy and MDD participants. This finding was consistent with the conceptualization that rumination acts as a maladaptive strategy in depressed individuals (Liverant et al., 2011). Related research showed that rumination can draw a person into negative thinking cycles and thus impairs mood and is associated with depressive symptoms (Lam et al., 2003). According to theories of ruminative response styles, individuals who take contemplation of depressive mood thinking style experience long and deep depressive symptoms (Nolen-Hoeksema, 1991). Furthermore, rumination mediated the association between neuroticism and depression in the current study. These results are consistent with and refined the findings of previous research (Muris et al., 2005; Kuyken et al., 2006; Roelofs et al., 2008). Highly neurotic individuals are sensitive to negative information and have a high tendency to experience negative effects when encountering stressors (Lahey, 2011). This negative experience is enhanced by rumination, thereby increasing depressive symptoms (Mclaughlin and Nolenhoeksema, 2011). A longitudinal cohort study suggested that rumination scores are not only related to current depressive symptoms but also predict the levels of depressive symptoms 12 months later (Wilkinson et al., 2013). These results suggest that rumination enhances the negative effect of neuroticism on depressive symptoms both in healthy and MDD people.

In the present study, thought control was negatively related to neuroticism and depression. This finding is in line with the result that weak thought control ability is positively related to neuroticism (Luciano et al., 2005) and indeed predictive of depressive symptoms (Peterson et al., 2009; Gotlib and Joormann, 2010; Williams et al., 2010). Importantly, current results revealed a new evidence that thought control mediates the association between neuroticism and depressive symptoms among healthy and MDD people. Previous studies support this evidence. Highly neurotic individuals are sensitive to negative information when encountering stress (Lahey, 2011). Thought control plays a key role in limiting negative information that enters the working memory and in removing irrelevant information from the working memory, which could help individuals avoid drawing rumination in negative thinking and have less depressive symptoms (Joormann and Gotlib, 2010). These results indicate that thought control decreases negative outcomes, including depressive symptoms, in highly neurotic individuals.

This study is the first to estimate simultaneously the IEs of rumination and thought control on the association between neuroticism and depressive symptoms in the same model. The results of the current study suggest that rumination increases the negative impact of neuroticism on depressive symptoms. However, thought control decreases the negative impact of neuroticism on depressive symptoms. These results are consistent with the previous suggestion that a negative relation exists between rumination and ability to suppress memory retrieval (Hertel, 2004; Dieler et al., 2014; Fawcett et al., 2014). Inhibition deficits are associated with increased rumination (Davis and Nolenhoeksema, 2000). Rumination is a negative response style in neuroticism that enhances depression (Roelofs et al., 2008). Highly neurotic individuals are sensitive to negative information and rumination (Lahey, 2011), such that an individual is drawn into negative thinking cycles, which enhances the negative effects on depressive emotion (Mclaughlin and Nolenhoeksema, 2011). Meanwhile, thought control allows individuals to remove unwanted intrusive thoughts (Luciano et al., 2005). High-level thought control can prevent depressed individuals from ruminating in a negative emotion, thus reducing the negative outcomes of neuroticism on depression (Joormann and Gotlib, 2010). Therefore, the current empirical study demonstrated that high-level thought control prevents depressive symptoms arising from rumination among highly neurotic individuals.

The current study has some limitations that should be addressed. First, the cross-sectional study was unable to draw conclusions on the cause–effect relations. Therefore, prospective longitudinal studies are needed to interpret this association. Second, the results of this study showed that rumination is a risky thinking style that increases the effect of neuroticism on depression, but thought control is a positive thinking style that reduces the effect of neuroticism on depression. Whether this difference may be reflected in clinical intervention would be interesting to explore in further studies. Third, the IE of rumination was not significant between neuroticism and depression among MDD patients as compared with the effect of thought control. This finding may be caused by the small participant size in this study. Therefore, results need to be replicated in a larger participant size in MDD patients in future studies.

## Conclusion

This study investigated two relatively contrary potential cognitive processes underlying neuroticism and depressive symptoms, namely, rumination and thought control, across healthy and MDD participants. Current findings indicated that rumination increases the risk of neuroticism for depression, but high-level thought control decreases the negative effect of neuroticism on depression. To the best of our knowledge, this study is the first to obtain evidence that thought control mediates the relationship between neuroticism and depression among healthy and MDD adults. These findings can serve as a reference to develop interventions for improving cognitive control ability, which could effectively reduce depressive symptoms among highly neurotic individuals.

## Author Contributions

Q-LZ and JQ directed this study. F–YL conducted the statistical analysis and wrote the entire manuscript, W-JY helped to modify the manuscript. All authors contributed to and have approved the final manuscript.

## Conflict of Interest Statement

The authors declare that the research was conducted in the absence of any commercial or financial relationships that could be construed as a potential conflict of interest.
